# The shadow of dark matter as a shadow of string theory: string origin of the dipole term

**DOI:** 10.1140/epjc/s10052-020-8106-4

**Published:** 2020-06-12

**Authors:** Rainer Dick

**Affiliations:** Department of Physics and Engineering Physics, University of Saskatchewan, 116 Science Place, Saskatoon, SK S7N 5E2 Canada

## Abstract

We point out that the Kalb–Ramond field can couple to the Ramond and Neveu–Schwarz fields of superstring theory in a way that can generate a coupling of the Kalb–Ramond field to dark matter dipole moments. Electroweak dipole dark matter could then arise from the Ramond sector of superstrings with low string scale $M_s\lesssim 120$ TeV.

## Introduction

After the confirmation of the Higgs particle as the capstone of the Standard Model, solving the persistent enigma of dark matter has become a major objective of modern particle physics. The completion of the Standard Model (SM) is a great example of successful collaboration between theory and experiment, and theoretical models are needed in the “beyond SM” era to assist with the search for dark matter in particle physics experiments and to provide ideas on what to look for.

It is an intriguing possibility that dark matter might provide a window into low energy string phenomenology, e.g. through the swampland conjecture leading to fading dark matter within a tower of light string states [1, 2], or through models where a low string scale [3, 4] implies phenomenologically acceptable dark axions or dark gauge bosons [5–8]. It has been pointed out recently [9] that electroweak dipole dark matter [10, 11] may open a window into low energy string phenomenology through the Kalb–Ramond field. The question of the relevant string scale was not addressed in Ref. [9] because no direct link between the dark matter coupling and mass to the string scale was established. However, it turns out that the Kalb–Ramond field can couple to the fermionic Ramond fields in such a way that a low energy coupling of the Kalb–Ramond field to dark fermions can be traced back to string theory. This then allows for an estimate of the maximal string scale for which perturbatively coupled dipole dark matter could be induced from string theory.

Kalb and Ramond had noticed that gauge interactions between strings can be described in analogy to electromagnetic interactions if the basic Nambu–Goto action is amended with a coupling term to an anti-symmetric tensor field [12]. We follow the conventions of Ref. [9] and denote the Kalb–Ramond field with $C_{\mu \nu }=-\,C_{\nu \mu }$ to avoid confusion with the electroweak $U_Y(1)$ field strength tensor $B_{\mu \nu }$. The action for strings coupling to the Kalb–Ramond field is

1 $$\begin{aligned} S_1= & {} -\,T_s\int d^2\sigma \sqrt{(\dot{X}\cdot X')^2-\dot{X}^2 X'^2} \nonumber \\&+\,\frac{\mu _s}{2}\int d^2\sigma \left( \dot{X}^\mu X'^\nu -\dot{X}^\nu X'^\mu \right) C_{\mu \nu }(X) \nonumber \\&+\,g_b \int d\tau \left[ \dot{X}^\mu \mathcal {B}_{\mu }(X)\right] _{\sigma =0}^{\sigma =\ell } \end{aligned}$$

Here $T_s$ is the string tension and $\mu _s$ is a string charge with the dimension of mass. The world sheet measure is $d^2\sigma =d\tau d\sigma =d\sigma ^+ d\sigma ^-/2$, where $\sigma ^\pm =\sigma \pm \tau$.

The string equations of motion which follow from (1) are invariant under the KR gauge symmetry

2 $$\begin{aligned} C_{\mu \nu }\rightarrow C_{\mu \nu }+\partial _\mu f_\nu -\partial _\nu f_\mu ,\quad \mathcal {B}_\mu \rightarrow \mathcal {B}_\mu +(\mu _s/g_b)f_\mu , \end{aligned}$$

and under the *U*(1) gauge transformation $\mathcal {B}_\mu \rightarrow \mathcal {B}_\mu +\partial _\mu f$, and these gauge symmetries are preserved through addition of a field theory action for the gauge fields,

3 $$\begin{aligned} S_2= & {} \int d^4x\left( -\,\frac{1}{6}C^{\mu \nu \rho }C_{\mu \nu \rho }-\frac{1}{4}\mathcal {B}^{\mu \nu }\mathcal {B}_{\mu \nu } \right. \nonumber \\&+\left. \frac{\mu _s}{2g_b}C^{\mu \nu }\mathcal {B}_{\mu \nu } -\frac{\mu _s^2}{4g_b^2}C^{\mu \nu }C_{\mu \nu }\right) , \end{aligned}$$

where

4 $$\begin{aligned} C_{\mu \nu \rho }=\partial _\mu C_{\nu \rho }+\partial _\nu C_{\rho \mu }+\partial _\rho C_{\mu \nu } \end{aligned}$$

are the components of the 3-form field strength $C_3=dC$ of the Kalb–Ramond field. This motivated the proposal in [9] to induce a $U_Y(1)$ portal to dark matter through the Lagrangian

5 $$\begin{aligned} \mathcal {L}_{BC\chi }= & {} \overline{\chi }\left( \mathrm {i}\gamma ^\mu \partial _\mu -m_\chi \right) \chi -\frac{1}{6}C^{\mu \nu \rho }C_{\mu \nu \rho } \nonumber \\&-\,\frac{1}{2}m^2_C C_{\mu \nu }C^{\mu \nu } -g_{BC}m_C B^{\mu \nu }C_{\mu \nu } \nonumber \\&-\,g_{C\chi }\overline{\chi }S^{\mu \nu }(a_m+\mathrm {i}a_e\gamma _5)\chi C_{\mu \nu }. \end{aligned}$$

Elimination of $C_{\mu \nu }$ for energies much smaller than $m_C$ generates electroweak dipole moments for the dark matter field $\chi$,

6 $$\begin{aligned} \mathcal {L}_{B\chi }= & {} \frac{g_{BC}g_{C\chi }}{m_C}B_{\mu \nu } \overline{\chi }S^{\mu \nu }(a_m+\mathrm {i}a_e\gamma _5)\chi \nonumber \\= & {} \frac{g_{BC}g_{C\chi }}{m_C}(F_{\mu \nu }\cos \theta -Z_{\mu \nu }\sin \theta ) \nonumber \\&\times \overline{\chi }S^{\mu \nu }(a_m+\mathrm {i}a_e\gamma _5)\chi . \end{aligned}$$

However, while the bosonic terms in (5) could be motivated through the couplings of string world sheets to Kalb–Ramond fields, the coupling of $C_{\mu \nu }$ to the dark fermions had to be postulated. The purpose of the present paper is to point out that the Kalb–Ramond field can couple to the Ramond [13] and Neveu–Schwarz [14] fields of superstrings in a way that does induce magnetic dipole couplings at low energies, thus lending more credibility to the proposal that direct search for electroweak dipole dark matter can provide a window into low energy string phenomenology.

The possible string origin of the coupling of the Kalb–Ramond field to dark dipole moments is introduced in Sect. 2 and conclusions are formulated in Sect. 3. The mapping between world-sheet spinors and half-differentials is reviewed in Appendix A, since it helps to understand the proposed coupling of the Kalb–Ramond field to the half-differentials $\Psi ^\mu _{\sqrt{\pm }}$. The Kalb–Ramond field in Lorentz gauge and in Coulomb gauge and its polarization is reviewed in Appendix B.

## String origin of magnetic dipole terms

We wish to provide a string explanation for the coupling of the Kalb–Ramond field to the dark fermion dipole moments in Eq. (5). This will require a coupling to the Ramond fields $\psi ^\mu (\tau ,\sigma )$ on the world sheet, which we write as components $\Psi ^\mu _{\sqrt{\pm }}(\tau ,\sigma )$ of half-differentials. The mapping between 2D spinors and half-differentials is reviewed in Appendix A.

The mapping should respect world-sheet and target space symmetries under coordinate and Lorentz transformations. Since 2D spinors are completely equivalent to half-differentials, and since we can only use $C_{\mu \nu }$ or $C_{\mu \nu \rho }$ for the coupling, the unique solution to this problem is

7 $$\begin{aligned} S_3= & {} g_s\int d\sigma ^+ d\sigma ^-\, C_{\mu \nu \rho }(X) \left( \Psi ^\mu _{\sqrt{+}}\Psi ^\nu _{\sqrt{+}}\partial _- X^\rho \right. \nonumber \\&-\left. \Psi ^\mu _{\sqrt{-}}\Psi ^\nu _{\sqrt{-}}\partial _+ X^\rho \right) , \end{aligned}$$

with a dimensionless coupling constant $g_s$. The minus sign is required by parity invariance of the world-sheet Lagrangian.

The coupling term (7) is in world-sheet spinor notation (with metric determinant $g\equiv g(\tau ,\sigma )$ and zweibein components $e^\alpha {}_{a}\equiv e^\alpha {}_a(\tau ,\sigma )$) given by

8 $$\begin{aligned} S_3=-\,g_s\int d^2\sigma \,\sqrt{-g}\, C_{\mu \nu \rho }(X) e^\alpha {}_{a}\overline{\psi }^\mu \gamma ^a\psi ^\nu \partial _\alpha X^\rho . \end{aligned}$$

The coupling (7) preserves the KR gauge symmetry $C\rightarrow C+df$ (2) because only the 3-form field strength *dC* (4) appears. However, for later reference it is also useful to explicitly note this on the level of the equations of motion. The contribution from (7) to the equation of motion of the Kalb–Ramond tensor is a source current

9 $$\begin{aligned} J_3^{\mu \nu }(x)= & {} -\,\frac{\delta S_3}{\delta C_{\mu \nu }(x)} =g_s\int d\sigma ^+ d\sigma ^-\,\frac{\partial }{\partial x^\rho }\delta (x-X) \nonumber \\&\times \left[ \Psi ^\rho _{\sqrt{+}}\Psi ^\mu _{\sqrt{+}}\partial _- X^\nu +\Psi ^\nu _{\sqrt{+}}\Psi ^\rho _{\sqrt{+}}\partial _- X^\mu \right. \nonumber \\&+\,\Psi ^\mu _{\sqrt{+}}\Psi ^\nu _{\sqrt{+}}\partial _- X^\rho -\Psi ^\rho _{\sqrt{-}}\Psi ^\mu _{\sqrt{-}}\partial _+ X^\nu \nonumber \\&-\left. \Psi ^\nu _{\sqrt{-}}\Psi ^\rho _{\sqrt{-}}\partial _+ X^\mu -\Psi ^\mu _{\sqrt{-}}\Psi ^\nu _{\sqrt{-}}\partial _+ X^\rho \right] . \end{aligned}$$

This current satisfies

10 $$\begin{aligned} \partial _\mu J_3^{\mu \nu }(x)=0 \end{aligned}$$

due to the anti-symmetry of the bracket in (9) under $\mu \leftrightarrow \rho$. Eq. (10) ensures that the addition $J^{\mu \nu }\rightarrow J^{\mu \nu }+J_3^{\mu \nu }$ to the source terms of the original Kalb–Ramond equations of motion complies with gauge invariance, see also Appendix B.

The couplings (1, 7) of the Kalb–Ramond field break the world-sheet supersymmetry of the free string theory. However, they do preserve the GSO projection [15], and that is what we really need. The couplings (1, 7) correspond to interactions between string world sheets which are mediated by a spacetime field. This leads to a Dyson representation for the scattering matrix,

11 $$\begin{aligned} S_{fi}=\langle f|\mathrm {T}\exp \left( -\,\mathrm {i}\int d^4x\,\mathcal {H}_I(x)\right) |i\rangle , \end{aligned}$$

with the Hamiltonian density in the interaction picture. The contribution to $\mathcal {H}_I$ from Eq. (7) is in Lorentz gauge^1^

12 $$\begin{aligned} \mathcal {H}_3(x)= & {} -\,g_s\int d\sigma ^+ d\sigma ^-\, C_{\mu \nu \rho }(x) \left( \Psi ^\mu _{\sqrt{+}}\Psi ^\nu _{\sqrt{+}}\partial _- X^\rho \right. \nonumber \\&-\left. \Psi ^\mu _{\sqrt{-}}\Psi ^\nu _{\sqrt{-}}\partial _+ X^\rho \right) \delta (x-X(\tau ,\sigma )). \end{aligned}$$

The Kalb–Ramond type interactions (1, 7) extend the sector of string interactions by introducing a spacetime field which probes the bosonic and fermionic string excitations across the world sheet. We could think of them in terms of vertex operator insertions on the world sheet, but they do not generate additional constraints from branch cuts. The consistent string theories are therefore still built from tensor products of the Ramond sectors $(\mathrm {R},\pm )$ and the Neveu–Schwarz sector $(\mathrm {NS},+)$ [16], where the signs denote the level of fermionic world sheet excitations modulo 2. The interactions (1, 7) can change the level of fermionic world sheet excitations only in even steps, and they cannot change periodicity conditions on closed strings or boundary counditions on open strings. Therefore they map states in a consistent string theory into states of that theory. As a consequence, and in keeping with the proposal of Kalb and Ramond of fundamental 1-branes interacting through exchange of fundamental 0-branes, the states of string theory with Kalb–Ramond type interactions are tensor products of string states in the Ramond or Neveu–Schwarz sectors of the theory with Kalb–Ramond states $|\varvec{k}_1,\ldots \varvec{k}_M;\varvec{p}_1,\ldots \varvec{p}_N,\alpha _1,\ldots \alpha _N\rangle$, where $\varvec{k}_I$ and $\varvec{p}_I$ are momenta of Kalb–Ramond tensor fields $C_{\mu \nu }$ and vector fields $\mathcal {B}_\mu$, respectively, and the $\alpha _I$ denote the polarizations of the vector fields.^2^ There are no polarization labels for the tensor field, because anti-symmetric tensor fields $C_{\mu \nu }$ with gauge symmetry and 3-momentum $\varvec{k}$ have only one possible physical polarization state in four dimensions, *viz.* $\epsilon ^{(1)}_\mu (\varvec{k})\epsilon ^{(2)}_\nu (\varvec{k}) -\varvec{\epsilon }^{(2)}_\mu (\varvec{k})\epsilon ^{(1)}_\nu (\varvec{k})$, where $k\cdot \epsilon ^{(\alpha )}(\varvec{k})=\varvec{k}\cdot \varvec{\epsilon }^{(\alpha )}(\varvec{k})=0$, i.e. the basis $\epsilon ^{(\alpha )}(\varvec{k})$ is like the polarization basis of physical states of a massless vector field with momentum $\varvec{k}$. However, in Lorentz gauge there are two additional unphysical polarizations of the form $\epsilon ^{(\alpha )}_\mu (\varvec{k})\epsilon ^{(3)}_\nu (\varvec{k}) -\epsilon ^{(3)}_\mu (\varvec{k})\epsilon ^{(\alpha )}_\nu (\varvec{k})$, $k\cdot \epsilon ^{(3)}(\varvec{k})=0\ne \varvec{k}\cdot \varvec{\epsilon }^{(3)}(\varvec{k})$, see also Appendix B.

If we evaluate the expression (7) on the low-energy states

13 $$\begin{aligned}&|\varvec{k},s\rangle =|0\rangle u(\varvec{k},s),\quad u^+(\varvec{k},s)\cdot u(\varvec{k},s')=\delta _{s,s'}, \end{aligned}$$

14 $$\begin{aligned}&\partial _\pm X^\mu \Rightarrow \pm k^\mu /2k^0, \quad \Psi ^\mu _{\sqrt{\pm }}\Rightarrow \mathrm {i}\gamma ^\mu /\sqrt{8}, \end{aligned}$$

of the Ramond sector, we find in temporal gauge $\tau =t$, $\int d\sigma =\ell =k^0/T_s$,

15 $$\begin{aligned} S_3= & {} \frac{g_s}{8}\int dt\, C_{\mu \nu \rho } \left( x+\frac{kt}{k^0}\right) \frac{k^\rho }{T_s} \nonumber \\&\times \overline{u}(\varvec{k},s)\cdot [\gamma ^\mu ,\gamma ^\nu ]\cdot u(\varvec{k},s). \end{aligned}$$

This describes the interaction of the Kalb–Ramond field with a classical fermion. The corresponding quantum mechanical action is

16 $$\begin{aligned} S_3=g_s\frac{\pi ^3}{\mathrm {i}T_s}\int d^4x\,C^{\mu \nu \rho }(x) \overline{\chi }(x)\cdot [\gamma _\mu ,\gamma _\nu ]\cdot \partial _\rho \chi (x). \end{aligned}$$

The non-relativistic limit of Eq. (16) corresponds to a coupling

17 $$\begin{aligned} S_3=-\,4g_s\frac{\pi ^3}{T_s}m_\chi m_C\int d^4x\,C^{ij}(x) \overline{\chi }(x) S_{ij}\chi (x), \end{aligned}$$

where we invoked the standard assumption that the low energy string states in the *a priori* massless sector acquire masses through symmetry breaking.

The coupling of the Kalb–Ramond field to fermions on the world-sheet (7) and in the resulting field theory coupling (16) are explicitly invariant under the KR gauge symmetry because the fermions couple to the Kalb–Ramond field strength. The resulting coupling (17) in the non-relativistic limit is effectively invariant within that limit due to $m_C\gg |\varvec{k}|$, where $\varvec{k}$ is the 3-momentum of the Kalb–Ramond field. We have $m_C |C_{ij}|\simeq |\partial _0 C_{ij}|\gg |\partial _i C_{0j}|$, and therefore $m_C C_{ij}\simeq \mathrm {i}C_{0ij}$.

Elimination of $C^{ij}(x)$ from the action with the terms (5) yields a magnetic dipole coupling with coupling constant

18 $$\begin{aligned} \frac{a_m}{M_d}=g_{BC}g_{C\chi }\frac{a_m}{m_C}=4g_sg_{BC}\frac{\pi ^3}{T_s}m_\chi . \end{aligned}$$

However, the coupling scale $M=M_d/a_m$ can be determined as a function of dark matter mass $m_\chi$ through the requirement of thermal dark matter creation in the early universe. This led to $M\simeq 23$ PeV for $m_\chi =1$ MeV and $M\simeq 3.7$ TeV for $m_\chi =10$ GeV, or roughly^3^ $m_\chi M\simeq 3\times 10^4\,\mathrm {GeV}^2$, see Fig. 1 in Ref. [9]. However, Eq. (18) relates this to the string scale,

19 $$\begin{aligned} T_s=4g_sg_{BC}\pi ^3 m_\chi M. \end{aligned}$$

String-induced electroweak dipole dark matter with perturbative couplings would therefore require a low string scale $\sqrt{8\pi T_s}\lesssim 120$ TeV.

## Conclusion

Inclusion of the missing piece (7) in the string derivation of electroweak dipole dark matter relates the string tension to dark matter parameters. Assuming perturbative couplings and that dark matter is created from thermal freeze-out then leads to the requirement $M_s\lesssim 120$ TeV. Since string theory appears to be unique in providing a mechanism to induce electroweak dipole moments in dark matter, discovery of electroweak dipole dark matter with a mass and recoil cross section which agrees with the predictions from thermal dark matter creation would be a strong hint for low-scale string theory.

It is intriguing that the geometry of string ground states allows for the construction of phenomenologically viable models with fundamental string scales all the way down to the TeV scale [3–8]. Furthermore, it is also conceivable that there may exist different incarnations of fundamental strings with different tensions $T_s$. In that case the Kalb–Ramond model (1) and its extension (7) could find a natural “pure string” explanation within a hierarchy of string scales $T_s\ll T_P=M^2_\mathrm{Planck}/8\pi$. The Kalb–Ramond field $C_{\mu \nu }(x)$ would then correspond to the low energy description of the anti-symmetric tensor fields of Planck scale superstrings with tension $T_P$, and the couplings (1, 7) would arise from world-sheet couplings between strings with different tensions. If the low scale string sector contains only Ramond sector states, such a scenario would not need to invoke any further assumption about the nature of the string ground state to explain why $M_\mathrm{Planck}\gg M_s$. Either way, the possibility to induce electroweak dipole dark matter from string theory adds to the anticipation that low scale string theory should become a leading paradigm for particle physics searches beyond the Standard Model, both in direct dark matter search experiments and at future colliders.

## Data Availability

This manuscript has no associated data or the data will not be deposited. [Authors’ comment: This manuscript has no associated data since no datasets were generated or analyzed during the current study].

## Appendix A: Half-differentials and fermions on the world sheet

Here we review the mapping between spinors and half-differentials on world sheets in the conformal gauge

20 $$\begin{aligned} g_{\tau \tau }+g_{\sigma \sigma }=\dot{X}^2+X'^2=0,\quad g_{\tau \sigma }=\dot{X}\cdot X'=0. \end{aligned}$$

We denote the remaining degree of freedom in the metric by $\phi (\tau ,\sigma )$,

21 $$\begin{aligned} g_{\sigma \sigma }=-\,g_{\tau \tau }=\exp (2\phi ). \end{aligned}$$

The metric in the corresponding two-dimensional light-cone coordinates $\sigma ^\pm =\sigma \pm \tau$,

22 $$\begin{aligned} g_{++}=g_{--}=0,\quad g_{+-}=\frac{1}{2}\exp (2\phi ) =\frac{1}{2}\sqrt{-g}, \end{aligned}$$

corresponds to zweibein components $e_\alpha {}^a$ (note $\eta _{+-}=1/2$),

23 $$\begin{aligned} e_+{}^+=e_-{}^-= & {} \exp (\phi ),\quad e_+{}^-=e_-{}^+=0, \end{aligned}$$

24 $$\begin{aligned} e_{-+}=e_{+-}= & {} \frac{1}{2}\exp (\phi ),\quad e_{++}=e_{--}=0. \end{aligned}$$

The set of coordinate transformations is restricted by the conformal gauge^4^ to

25 $$\begin{aligned} \sigma ^+\rightarrow \sigma '^+(\sigma ^+),\quad \sigma ^-\rightarrow \sigma '^-(\sigma ^-). \end{aligned}$$

On the other hand, the symmetry group *SO*(1, 1) of tangent plane Lorentz boosts also factorizes in the light-cone basis of tangent vectors. A boost with parameter

26 $$\begin{aligned} u=\mathrm {artanh}(\beta ) =\frac{1}{2}\ln \left( \frac{1+\beta }{1-\beta }\right) \end{aligned}$$

in the tangent plane yields in the light-cone basis $v^\pm =v^1\pm v^0$ the transformation law

27 $$\begin{aligned} \left( \begin{array}{c} v'^+\\ v'^-\\ \end{array} \right) =\left( \begin{array}{cc} \Lambda ^+{}_+ &{} 0\\ 0 &{} \Lambda ^-{}_- \end{array} \right) \left( \begin{array}{c} v^+\\ v^-\\ \end{array} \right) . \end{aligned}$$

with components

28 $$\begin{aligned} \Lambda ^+{}_+=\left( \frac{1-\beta }{1+\beta }\right) ^{1/2},\quad \Lambda ^-{}_-=\left( \frac{1+\beta }{1-\beta }\right) ^{1/2}. \end{aligned}$$

This is the exact square of the transformation of the spinor components in Weyl representation. Every Weyl representation of 2D $\gamma$ matrices yields either $\mathrm {i}S_{10}$ or $-\,\mathrm {i}S_{10}$ as the spinor representation of the boost generator, where

29 $$\begin{aligned} \mathrm {i}S_{10}=\frac{1}{2}\gamma _0\cdot \gamma _1 =\frac{\mathrm {1}}{2}\left( \begin{array}{cc} -1 &{} \,\,\,\,0 \\ \,\,\,\,0 &{} \,\,\,\,1 \\ \end{array}\right) . \end{aligned}$$

The corresponding Lorentz boost on the spinors is

30 $$\begin{aligned} \left( \begin{array}{c} \psi '^{\sqrt{+}}\\ \psi '^{\sqrt{-}}\\ \end{array} \right)= & {} \left( \begin{array}{cc} \exp (-\,u/2) &{} 0\\ 0 &{} \exp (u/2)\\ \end{array} \right) \left( \begin{array}{c} \psi ^{\sqrt{+}}\\ \psi ^{\sqrt{-}}\\ \end{array} \right) \nonumber \\= & {} \left( \begin{array}{cc} \left( \frac{1-\beta }{1+\beta }\right) ^{1/4} &{} 0\\ 0 &{} \left( \frac{1+\beta }{1-\beta }\right) ^{1/4} \end{array} \right) \left( \begin{array}{c} \psi ^{\sqrt{+}}\\ \psi ^{\sqrt{-}}\\ \end{array} \right) , \end{aligned}$$

i.e. the matrix elements of the spinor representation relate to the matrix elements of the vector representation through

31 $$\begin{aligned} \Lambda ^{\sqrt{\pm }}{}_{\sqrt{\pm }}=\sqrt{\Lambda ^{\pm }{}_{\pm }}. \end{aligned}$$

This motivates the assignments of indices to the components of the 2D Dirac spinor in the Weyl basis: $(\psi ^{\sqrt{+}})^2$ and $(\psi ^{\sqrt{-}})^2$ transform like the components of a tangent vector in the light-cone basis.

To be specific we use in the following calculations the real Weyl representation of 2D $\gamma$ matrices

32 $$\begin{aligned} \gamma ^0=\left( \begin{array}{cc} \,\,\,\,\,0 &{} -1\\ -1 &{} \,\,\,\,\,0\\ \end{array} \right) ,\quad \gamma ^1=\left( \begin{array}{cc} \,\,\,\,\,0 &{} \,\,\,\,1\\ -1 &{} \,\,\,\,0\\ \end{array} \right) . \end{aligned}$$

However, the mapping between world-sheet spinors and half-differentials, and the fermion action in terms of half-differentials, follow in the same form for every Weyl representation.

The tangent plane gamma matrices in the light cone basis follow as

33 $$\begin{aligned} \gamma ^+=-\,2\left( \begin{array}{cc} 0 &{} \,\,\,\,0\\ 1 &{} \,\,\,\,0\\ \end{array} \right) , \quad \gamma ^-=2\left( \begin{array}{cc} 0 &{} \,\,\,\,1\\ 0 &{} \,\,\,\,0\\ \end{array} \right) , \end{aligned}$$

and the transformation of the integration measure to the light-cone coordinates on the world sheet is

34 $$\begin{aligned} d\tau d\sigma \sqrt{-g}=d\sigma ^+ d\sigma ^- g_{+-}. \end{aligned}$$

The curved space generalization of the kinetic terms in the Dirac action is

35 $$\begin{aligned}&\frac{\mathrm {i}}{2} e^\alpha {}_a \left[ (\overline{\psi }\Omega _\alpha -\partial _\alpha \overline{\psi })\gamma ^a\psi +\overline{\psi }\gamma ^a (\partial _\alpha \psi +\Omega _\alpha \cdot \psi )\right] \nonumber \\&=\frac{\mathrm {i}}{2}e^\alpha {}_a \left[ \overline{\psi }\gamma ^a\partial _\alpha \psi -\partial _\alpha \overline{\psi }\cdot \gamma ^a\psi +\overline{\psi }\{\Omega _\alpha ,\gamma ^a\}\psi \right] . \end{aligned}$$

However, the spin-connection in two dimensions,

36 $$\begin{aligned} \Omega _\alpha =-\,\mathrm {i}\Gamma ^0{}_{1\alpha }S^1{}_0 =\frac{1}{2}\Gamma ^0{}_{1\alpha }\gamma ^1\gamma _0, \end{aligned}$$

anti-commutes both with $\gamma ^0$ and $\gamma ^1$, $\{\Omega _\alpha ,\gamma ^a\}=0$. Therefore, splitting the derivative symmetrically between $\overline{\psi }$ and $\psi$ cancels the spin connection from the Dirac action in two dimensions. The resulting action combining the Fubini-Veneziano fields and Dirac spinors on the world sheet is

37 $$\begin{aligned} S= & {} -\int d\tau \int _0^{\ell } d\sigma \,\sqrt{-g}\left[ \frac{T_s}{2} g^{\alpha \beta }\partial _\alpha X\cdot \partial _\beta X\right. \nonumber \\&+\left. \frac{\mathrm {i}}{2}\sqrt{T_s}e^\alpha {}_a\left( \partial _\alpha \overline{\psi }\cdot \gamma ^a\cdot \psi -\overline{\psi }\cdot \gamma ^a\cdot \partial _\alpha \psi \right) \right] \nonumber \\= & {} -\int d\sigma ^+ d\sigma ^- g_{+-}\Big [T_s g^{+-}\partial _+ X\cdot \partial _- X \nonumber \\&+\,\frac{\mathrm {i}}{2}\sqrt{T_s}e^+{}_+\left( \partial _+\overline{\psi }\cdot \gamma ^+\cdot \psi -\overline{\psi }\cdot \gamma ^+\cdot \partial _+\psi \right) \nonumber \\&+\,\frac{\mathrm {i}}{2}\sqrt{T_s}e^-{}_-\left( \partial _-\overline{\psi }\cdot \gamma ^-\cdot \psi -\overline{\psi }\cdot \gamma ^-\cdot \partial _-\psi \right) \Big ]. \end{aligned}$$

Substituting the spinor components $\psi =(\psi ^{\sqrt{+}},\psi ^{\sqrt{-}})$, $\overline{\psi }=(-\,\psi ^{\sqrt{-},*},-\,\psi ^{\sqrt{+},*})$, and the $\gamma$-matrices (33) yields

38 $$\begin{aligned} S= & {} -\int d\sigma ^+ d\sigma ^-\Big [ T_s\partial _+ X\cdot \partial _- X \nonumber \\&+\,\mathrm {i}\sqrt{T_s}e_{-+}\left( \partial _+\psi ^{\sqrt{+},*}\cdot \psi ^{\sqrt{+}} -\psi ^{\sqrt{+},*}\cdot \partial _+\psi ^{\sqrt{+}}\right) \nonumber \\&+\,\mathrm {i}\sqrt{T_s}e_{+-}\left( \psi ^{\sqrt{-},*}\cdot \partial _-\psi ^{\sqrt{-}}- \partial _-\psi ^{\sqrt{-},*}\cdot \psi ^{\sqrt{-}}\right) \Big ] \nonumber \\= & {} - \int d\sigma ^+ d\sigma ^-\Big [ T_s\partial _+ X\cdot \partial _- X \nonumber \\&+\,\mathrm {i}\sqrt{T_s} \partial _+\left( \sqrt{e_{-+}}\psi ^{\sqrt{+},*}\right) \cdot \left( \sqrt{e_{-+}}\psi ^{\sqrt{+}}\right) \nonumber \\&-\,\mathrm {i}\sqrt{T_s}\left( \sqrt{e_{-+}}\psi ^{\sqrt{+},*}\right) \cdot \partial _+\left( \sqrt{e_{-+}}\psi ^{\sqrt{+}}\right) \nonumber \\&+\,\mathrm {i}\sqrt{T_s}\left( \sqrt{e_{+-}}\psi ^{\sqrt{-},*}\right) \cdot \partial _-\left( \sqrt{e_{+-}}\psi ^{\sqrt{-}}\right) \nonumber \\&-\,\mathrm {i}\sqrt{T_s}\partial _-\left( \sqrt{e_{+-}}\psi ^{\sqrt{-},*}\right) \cdot \left( \sqrt{e_{+-}}\psi ^{\sqrt{-}}\right) \Big ]. \end{aligned}$$

Combining the spinor and zweibein components yields the half-differentials

39 $$\begin{aligned} \Psi _{\sqrt{-}}(\xi )= & {} \sqrt{e_{-+}}(\xi )\psi ^{\sqrt{+}}(\xi ), \end{aligned}$$

40 $$\begin{aligned} \Psi _{\sqrt{+}}(\xi )= & {} \sqrt{e_{+-}}(\xi )\psi ^{\sqrt{-}}(\xi ), \end{aligned}$$

and the action

41 $$\begin{aligned} S= & {} - \int d\sigma ^+ d\sigma ^-\Big [T_s\partial _+ X\cdot \partial _- X \nonumber \\&+\,\mathrm {i}\sqrt{T_s} \Big (\Psi _{\sqrt{+}}^*\cdot \partial _-\Psi _{\sqrt{+}} -\partial _-\Psi _{\sqrt{+}}^*\cdot \Psi _{\sqrt{+}}\Big ) \nonumber \\&+\,\mathrm {i}\sqrt{T_s} \Big (\partial _+\Psi _{\sqrt{-}}^*\cdot \Psi _{\sqrt{-}} -\Psi _{\sqrt{-}}^*\cdot \partial _+\Psi _{\sqrt{-}}\Big )\Big ]. \end{aligned}$$

The 2D metric has completely disappeared, in the scalar sector due to the conformal gauge and in the spinor sector due to absorption into the half-differentials.

The spinors are invariant under coordinate transformations on the world sheet and transform under Lorentz transformations in the tangent plane according to (30). The half-differentials (39,40) are invariant under Lorentz transformations, but transform under the coordinate transformations (25) according to

42 $$\begin{aligned} \Psi '_{\sqrt{-}}(\xi ')= & {} \Psi _{\sqrt{-}}(\xi ) \sqrt{d\sigma ^-/d\sigma '^-}, \end{aligned}$$

43 $$\begin{aligned} \Psi '_{\sqrt{+}}(\xi ')= & {} \Psi _{\sqrt{+}}(\xi ) \sqrt{d\sigma ^+/d\sigma '^+}. \end{aligned}$$

The phases of the anti-commuting half-differentials decouple in the action (41), in agreement with the real transformation behavior (30) of the spinors in Weyl representation. The standard phase convention is

44 $$\begin{aligned} \Psi _{\sqrt{\pm }}^{\mu *}=\Psi _{\sqrt{\pm }}^\mu . \end{aligned}$$

The superconformal transformation parameters $\epsilon ^{\sqrt{\pm }}(\sigma ^\pm )$ are then real anti-commuting $(-1/2)$-differentials^5^, which yield the transformations

45 $$\begin{aligned} \delta X^\mu= & {} \frac{\mathrm {i}}{\sqrt{T_s}}\left( \epsilon ^{\sqrt{+}}\Psi ^\mu _{\sqrt{+}} +\epsilon ^{\sqrt{-}}\Psi ^\mu _{\sqrt{-}}\right) , \end{aligned}$$

46 $$\begin{aligned} \delta \Psi ^\mu _{\sqrt{+}}= & {} -\,\frac{1}{2} \epsilon ^{\sqrt{+}}\partial _+ X^\mu ,\quad \delta \Psi ^\mu _{\sqrt{-}}=\frac{1}{2} \epsilon ^{\sqrt{-}}\partial _- X^\mu . \end{aligned}$$

## Appendix B: The Kalb–Ramond field in Lorentz gauge and in Coulomb gauge, and its polarizations

We used Lorentz gauge for the discussion of the preservation of GSO projection sectors in Sect. 2. Lorentz gauge is also preferred for covariant perturbation theory and therefore we also take this opportunity to point out that it can be imposed simultaneously on both $C_{\mu \nu }$ and $\mathcal {B}_\mu$. After imposing $\partial ^\mu \mathcal {B}_\mu =0$ in the standard way using the *U*(1) gauge symmetry of the vector field, we can use a KR gauge transformation $C^{(0)}_{\mu \nu }\rightarrow C_{\mu \nu }$ with the KR gauge function

47 $$\begin{aligned} f_\nu (x)=\int d^4x' G(x-x')\partial '^\mu C^{(0)}_{\mu \nu }(x') \end{aligned}$$

to impose $\partial ^\mu C_{\mu \nu }(x)=0$ while maintaining $\partial ^\mu \mathcal {B}_\mu =0$. The Green function is the retarded function

48 $$\begin{aligned} G(x)=\frac{1}{4\pi |\varvec{x}|}\delta (t-|\varvec{x}|),\quad \partial ^2G(x)=-\,\delta (x). \end{aligned}$$

This preserves $\partial ^\mu \mathcal {B}_\mu =0$ due to

49 $$\begin{aligned} \partial ^\nu f_\nu (x)= & {} \int d^3\varvec{x}'\left. \frac{1}{4\pi |\varvec{x}-\varvec{x}'|} \partial '^\mu \partial '^\nu C^{(0)}_{\mu \nu }(x')\right| _{t'=t-|\varvec{x}-\varvec{x}'|}\nonumber \\= & {} 0, \end{aligned}$$

and ensures that $C_{\mu \nu }(x)=C^{(0)}_{\mu \nu }(x)+\partial _\mu f_\nu (x)-\partial _\nu f_\mu (x)$ satisfies $\partial ^\mu C_{\mu \nu }(x)=0$.

On the other hand, the dominant contribution to electromagnetic interactions between low-velocity charged particles can be expressed in Coulomb gauge through Coulomb potentials. In string theory with Kalb–Ramond interactions, Coulomb gauge is useful to describe the impact of Kalb–Ramond exchange on string-string interaction forces. Therefore it is also useful to know that KR gauge symmetry can be used to impose a Coulomb gauge condition $\partial ^iC_{i\mu }=0$ for the tensor field while preserving Coulomb gauge for the vector field. The KR gauge function

50 $$\begin{aligned} f_\nu (\varvec{x},t)=\int d^3\varvec{x}'\,\frac{1}{4\pi |\varvec{x}-\varvec{x}'|}\partial '^i C^{(0)}_{i\nu }(\varvec{x}',t) \end{aligned}$$

accomplishes this due to $\partial ^i f_i(x)=0$.

To determine the polarizations of the Kalb–Ramond tensor in the interaction picture states, we note that

51 $$\begin{aligned} \partial _\mu C^{\mu \nu \rho }-m_C^2 C^{\nu \rho }=-\,J^{\nu \rho }, \end{aligned}$$

where the currents $J^{\nu \rho }$ satisfy the consistency condition

52 $$\begin{aligned} \partial _\nu J^{\nu \rho }=m_C^2\partial _\nu C^{\nu \rho } \end{aligned}$$

as a consequence of the contributions of the vector field $\mathcal {B}_\mu$ which maintains the KR gauge symmetry in the presence of the mass term for $C_{\mu \nu }$, see Eqs. (10–13) in Ref. [9] and Eqs. (9, 10) in Sect. 2. The relevant mode expansions of the interaction picture field operators therefore still contain only one physical polarization state [18]. Coulomb’s law and the mode expansion for the remaining degrees of freedom in Coulomb gauge are

53 $$\begin{aligned} (\Delta -m_C^2)C^{0k}=-\,J^{0k} \end{aligned}$$

and

54 $$\begin{aligned} C_{ij}(x)= & {} \int \frac{d^3\varvec{k}}{4\sqrt{\pi ^3E(\varvec{k})}}\,\hat{k}^h\epsilon _{hij} [a(\varvec{k})\exp (\mathrm {i}k\cdot x) \nonumber \\&+\,a^+(\varvec{k})\exp (-\,\mathrm {i}k\cdot x)], \end{aligned}$$

respectively, whereas the mode expansion in Lorentz gauge is

55 $$\begin{aligned} C_{\mu \nu }(x)= & {} \int \frac{d^3\varvec{k}}{4\sqrt{\pi ^3E(\varvec{k})}}\,\epsilon _{\alpha \beta \gamma } \epsilon ^{(\beta )}_\mu (\varvec{k})\epsilon ^{(\gamma )}_\nu (\varvec{k}) \nonumber \\&\times [a^{(\alpha )}(\varvec{k})\exp (\mathrm {i}k\cdot x) \nonumber \\&+\,a^{(\alpha )+}(\varvec{k})\exp (-\,\mathrm {i}k\cdot x)]. \end{aligned}$$

We choose polarization vectors $\epsilon ^{(\alpha )}_\mu (\varvec{k})$ such that for $\alpha \in \{1,2\}$

56 $$\begin{aligned} k\cdot \epsilon ^{(\alpha )}(\varvec{k})=\varvec{k}\cdot \varvec{\epsilon }^{(\alpha )}(\varvec{k})=0, \end{aligned}$$

whereas $$\epsilon _0^{(3)}(\varvec{k})\ne 0$$,

57 $$\begin{aligned} k\cdot \epsilon ^{(3)}(\varvec{k})=0\ne \varvec{k}\cdot \varvec{\epsilon }^{(3)}(\varvec{k}). \end{aligned}$$

Comparison with the operator (54) in Coulomb gauge shows that the single transverse physical polarization in Lorentz gauge is $\epsilon ^{(1)}_\mu (\varvec{k})\epsilon ^{(2)}_\nu (\varvec{k}) -\epsilon ^{(2)}_\mu (\varvec{k})\epsilon ^{(1)}_\nu (\varvec{k})$,

58 $$\begin{aligned}{}[\varvec{\epsilon }^{(1)}(\varvec{k})\otimes \varvec{\epsilon }^{(2)}(\varvec{k}) -\varvec{\epsilon }^{(2)}(\varvec{k})\otimes \varvec{\epsilon }^{(1)}(\varvec{k})]\cdot \varvec{k}=0, \end{aligned}$$

whereas the two spatially longitudinal polarizations,

59 $$\begin{aligned}{}[\varvec{\epsilon }^{(\alpha )}(\varvec{k})\otimes \varvec{\epsilon }^{(3)}(\varvec{k}) -\varvec{\epsilon }^{(3)}(\varvec{k})\otimes \varvec{\epsilon }^{(\alpha )}(\varvec{k})]_{\alpha \in \{1,2\}} \cdot \varvec{k}\ne 0, \end{aligned}$$

are unphysical. Therefore the physical states of the Kalb–Ramond tensor,

60 $$\begin{aligned} |\varvec{k}_1,\ldots \varvec{k}_N\rangle =a^{(3)+}(\varvec{k}_1)\ldots a^{(3)+}(\varvec{k}_N)|0\rangle , \end{aligned}$$

look like spin-0 states. However, it is not possible to consistently replace $C_{\mu \nu }$ in interaction terms through a duality transformation into an axion. At the classical level, a relation of the form $C_{\mu \nu \rho }=\epsilon _{\mu \nu \rho \sigma }\partial ^\sigma a$ would require both fields to be massless free fields, and even if the equation would be consistent at the classical level, it would not constitute a canonical transformation of quantum operators.

## Footnotes

1 Lorentz gauge is not essential, of course, but convenient. Just like in QED, it yields the Lagrangian interaction terms as covariant vertices. It can be imposed on $C^{\mu \nu }$ using the KR gauge symmetry (2) while preserving Lorentz gauge for $\mathcal {B}_\mu$, see Appendix B.

2 There is also another possibility. In multi-scale string theories with different kinds of strings with different tensions $T_s$, $T_{s'}$, the Kalb–Ramond picture might correspond to an effective intermediate energy theory for energies $T_s\lesssim E\ll T_{s'}$, such that for the high scale string modes an effective field theory description applies, whereas for the low-scale strings the world sheet description is still needed. In that case, the Kalb–Ramond field could arise from the field theory description of the anti-symmetric tensor modes of the high tension string theory. The states in the intermediate energy effective theory would still have the form described in the text, but the Kalb–Ramond factors would arise from underlying string states in the high-tension string theory.

3 This is a generic feature of thermal dark matter creation. Larger dark matter mass requires stronger coupling for later freeze-out, since larger $m_\chi$ requires fewer dark matter particles to explain the observed dark matter abundance.
